# 66 Key Features of Virtual Reality Impact Effectiveness of Pain Reduction in Pediatric Burn Patients

**DOI:** 10.1093/jbcr/irad045.040

**Published:** 2023-05-15

**Authors:** Henry Xiang, Megan Armstrong, Rajan Thakkar, Renata Fabia, Dana Noffsinger, Ai Ni, Jonathan Groner

**Affiliations:** Nationwide Children's Hospital, Columbus, Ohio; Nationwide Children's Hospital, Columbus, Ohio; Nationwide Children's Hospital, Columbus, Ohio; Nationwide Children's Hospital, Columbus, Ohio; Nationwide Children's Hospital, Columbus, Ohio; The Ohio State University, Columbus, Ohio; Nationwide Children's Hospital, Columbus, Ohio; Nationwide Children's Hospital, Columbus, Ohio; Nationwide Children's Hospital, Columbus, Ohio; Nationwide Children's Hospital, Columbus, Ohio; Nationwide Children's Hospital, Columbus, Ohio; Nationwide Children's Hospital, Columbus, Ohio; The Ohio State University, Columbus, Ohio; Nationwide Children's Hospital, Columbus, Ohio; Nationwide Children's Hospital, Columbus, Ohio; Nationwide Children's Hospital, Columbus, Ohio; Nationwide Children's Hospital, Columbus, Ohio; Nationwide Children's Hospital, Columbus, Ohio; Nationwide Children's Hospital, Columbus, Ohio; The Ohio State University, Columbus, Ohio; Nationwide Children's Hospital, Columbus, Ohio; Nationwide Children's Hospital, Columbus, Ohio; Nationwide Children's Hospital, Columbus, Ohio; Nationwide Children's Hospital, Columbus, Ohio; Nationwide Children's Hospital, Columbus, Ohio; Nationwide Children's Hospital, Columbus, Ohio; The Ohio State University, Columbus, Ohio; Nationwide Children's Hospital, Columbus, Ohio; Nationwide Children's Hospital, Columbus, Ohio; Nationwide Children's Hospital, Columbus, Ohio; Nationwide Children's Hospital, Columbus, Ohio; Nationwide Children's Hospital, Columbus, Ohio; Nationwide Children's Hospital, Columbus, Ohio; The Ohio State University, Columbus, Ohio; Nationwide Children's Hospital, Columbus, Ohio; Nationwide Children's Hospital, Columbus, Ohio; Nationwide Children's Hospital, Columbus, Ohio; Nationwide Children's Hospital, Columbus, Ohio; Nationwide Children's Hospital, Columbus, Ohio; Nationwide Children's Hospital, Columbus, Ohio; The Ohio State University, Columbus, Ohio; Nationwide Children's Hospital, Columbus, Ohio; Nationwide Children's Hospital, Columbus, Ohio; Nationwide Children's Hospital, Columbus, Ohio; Nationwide Children's Hospital, Columbus, Ohio; Nationwide Children's Hospital, Columbus, Ohio; Nationwide Children's Hospital, Columbus, Ohio; The Ohio State University, Columbus, Ohio; Nationwide Children's Hospital, Columbus, Ohio

## Abstract

**Introduction:**

Virtual reality (VR) is a safe and effective pain management during burn dressing. However, key features of VR that impact the effectiveness of pain reduction remains unknown.

**Methods:**

Our randomized clinical trial included 90 children aged 6 to 17 years who were treated in the outpatient clinic of an American Burn Association–verified pediatric burn center. Active VR participants (n=31) played a VR game while passive VR participants (n=30) were immersed in the same VR environment without interactions. VR groups were also compared with a standard care group (n=29). The primary outcome was self-reported overall pain and worst pain during burn dressing using a visual analog scale (VAS; range, 0-100). Participants in the active and passive VR groups reported 3 key features of the VR game (engaging, fun, realistic) and all participants (VR and standard care group) reported how much time he/she thought about pain during the burn dressing. Key features of the VR game and time thinking about pain were reported using a scale ranging from 0 (not at all) to 100 (very much). A composite score was created by ranking scores of the 3 key features of the VR game and then summarizing them into one final composite score. Our study hypothesis was that the key features of the VR game significantly impact the effectiveness of VR in reducing pain during burn dressing care.

**Results:**

Nonparametric Spearman correlation analysis indicated the active VR group reported statistically significant higher average scores for all 3 key features of VR (engaging: 78.9, 95% confidence interval (CI)=68.5-89.3; fun: 85.7, 95%CI=76.6-94.7; realistic: 73.1, 95% CI=62.3-83.8) in comparison with the passive VR group (engaging: 72.7, 95% CI=59.9-85.5; fun: 77.3, 95%CI=65.6-89.0; realistic: 59.1, 95% CI=44.7-73.5) (all p-value < 0.01). The composite VR score was also significantly correlated with how much time the participant thought about pain during the burn dressing (coefficient= -0.344, p=0.007). The final multivariate logistic regression model of reporting moderate-to-severe pain found that key features of the VR game were statistically significant factors for overall pain and worst pain after adjusting for age, race/ethnicity, healing degree, pain medication use within 6 hours, and anxiety score (p< 0.01).

**Conclusions:**

This study provides evidence that key features of VR significantly impact its effectiveness in pain management among pediatric burn patients.

**Applicability of Research to Practice:**

Active VR game is more effective and should be used for pediatric pain management.

